# Expression of Endothelin-1 and Endothelial Nitric Oxide Synthase in Normal and Preeclamptic Placentae

**DOI:** 10.1055/s-0042-1742317

**Published:** 2022-02-25

**Authors:** Aung Khaing, Aye Thet Swe, Cho Lwin Aung, Mya Mya Thwin, Mya Thanda Sein

**Affiliations:** 1 Department of Physiology, University of Medicine 2, Yangon, The Republic of the Union of Myanmar; 2 Physiology Unit, Universiti Sultan Zanial Abidin, Kuala Terengganu,Terengganu, Malaysia

**Keywords:** endothelial nitric oxide synthase, endothelin-1, placenta, preeclampsia, óxido nítrico sintase endotelial, endotelina-1, placenta, pré-eclâmpsia

## Abstract

**Objective** To investigate the expression of endothelin-1 (ET-1) and endothelial nitric oxide (NO) synthase (eNOS) in normal and preeclamptic (PE) placentae.

**Methods** The present cross-sectional analytical study was performed in normal and PE primigravidae ( *n* = 10 in each group) who were admitted to the North Okkalapa General and Teaching Hospital from February 2019 to February 2020. Serum samples were collected immediately before delivery, and placental tissues were collected immediately after emergency or elective cesarean section. The expression of placental eNOS was measured by western blot, and the levels of ET-1 in placental tissue homogenates and in the serum were measured by enzyme-linked immunosorbent assay (ELISA).

**Results** The PE group had significantly higher serum levels of ET-1 (median: 116.56 pg/mL; IQR: 89.14–159.62 pg/mL) than the normal group (median: 60.02 pg/mL; IQR: 50.89–94.37 pg/mL) ( *p* < 0.05). However, statistically significant differences were not observed in the levels of ET-1 in placental tissue homogenates between normal and PE placentae (median: 0.007 pg/µg of total protein; IQR: 0.002–0.0123 pg/µg of total protein; and median: 0.005 pg/µg of total protein; IQR: 0.003–0.016 pg/µg of total protein respectively). The median and IQR values of relative placental eNOS expression were significantly higher in the PE group than in the normal group ( *p* < 0.05). The serum levels of ET-1 level were not significantly correlated with placental ET-1 expression, and neither there was a significant correlation between placental ET-1 and eNOS expression in any of the groups.

**Conclusion** The serum levels of ET-1 were significantly higher in PE pregnant women compared with normal pregnant women, while the ET-1 levels of placental tissue homogenates were not significantly different. Serum ET-1 rather than placental ET-1 might play a major role in the pathogenesis of PE.

## Resumo

**Objetivo** Investigar a expressão da endotelina-1 (ET-1) e da óxido nítrico sintase endotelial ( *endothelial nitric oxide synthase* , eNOS, em inglês) em placentas normais e com pré-eclâmpsia (PE).

**Métodos** Este estudo transversal e analítico foi realizado em primigestas normais e com PE ( *n* = 10 em cada grupo) internadas no Hospital Geral e das Clínicas de North Okkalapa entre fevereiro de 2019 e fevereiro de 2020. Amostras de soro foram coletadas imediatamente antes do parto, e os tecidos placentários foram coletados imediatamente após cesariana de emergência ou eletiva. A expressão da eNOS da placenta foi medida por *western blot* , e os níveis de ET-1 nos homogenatos de tecidos placentários ET-1 e no soro foram medidos por ensaio de imunoabsorção enzimática.

**Resultados** O grupo com PE apresentou níveis séricos de ET-1 significativamente mais altos (mediana: 116,56 pg/mL; IIQ: 89,14–159,62 pg/mL) do que o grupo normal (mediana: 60,02 pg/mL; IIQ: 50,89–94,37 pg/mL) ( *p* < 0,05). No entanto, não foi observada diferença significativa no nível ET-1 dos homogenatos de tecidos placentários entre placentas normais e com PE (mediana: 0,007 pg/µg de proteína total; IIQ: 0,002–0,0123 pg/µg de proteína total; e mediana: 0,005 pg/µg de proteína total; IIQ: 0,003–0,016 pg/µg de proteína total, respectivamente). Os valores da mediana e do IIQ da expressão relativa da eNOS placentária foram significativamente maiores no grupo com PE do que no grupo normal ( *p* < 0,05). O nível sérico de ET-1 não estava significativamente correlacionado com a expressão placentária de ET-1, e tampouco houve correlação significativa entre as expressões placentária de ET-1 e de eNOS em nenhum dos grupos.

**Conclusão** Os níveis séricos de ET-1 foram significativamente maiores em gestantes com PE em comparação com gestantes normais, ao passo que os níveis de ET-1 dos homogenatos de tecidos placentários não foram significativamente diferentes. A ET-1 sérica, em vez da ET-1 placentária, pode desempenhar um papel importante na patogênese da EP.

## Introduction

The central organ in the pathogenesis of preeclampsia (PE) is the placenta, and the only definitive cure is delivery. Abnormal placentation and endothelial dysfunction play a key role in the pathological changes in PE. Nitric oxide (NO) and endothelin-1 (ET-1) are natural counterparts in vascular function, and an imbalance between these two mediators is a characteristic of endothelial dysfunction and is important in the progression of PE. 1

Nitric oxide is synthesized from L-arginine, and it is catalyzed by a key enzyme called endothelial nitric oxide synthase (eNOS), 2 and studies have investigated the association between the expression of placental eNOS and PE. In an in vitro study, Baker et al. 3 observed that eNOS activity was significantly increased in endothelial cells exposed to the plasma of preeclamptic women. Likewise, Smith-Jackson et al. 4 observed a significant increase in the mRNA expression of placental eNOS in preeclamptic pregnant women compared with normal pregnant women. However, Wang et al. 5 observed a significant reduction of eNOS expression in cultured endothelial cells from preeclamptic pregnant women compared with normal pregnant women. Accordingly, these associations are still controversial, and will remain the subject of active investigation.

Evidence from clinical studies suggest that serum ET-1 is a final common pathway in the pathophysiology of PE 6 7 8 9 10 which indicates the positive correlation between the level of circulating ET-1 and the severity of disease symptoms. 11 However, there are still controversial findings concerning the level of placental ET-1 in normal pregnant women and those with PE. Studies have found that the levels of tissue endothelin in placental tissues were higher, 12 13 lower, 14 or not significantly different 8 15 16 in preeclamptic pregnant women compared with normal pregnant women.

In the vessels, NO inhibits the bioavailability of ET-1 under physiological conditions. 1 When NO is diminished, it cannot inhibit the activity of ET-1. Consequently, unmitigated ET-1 regulates itself through a negative-feedback mechanism by stimulating eNOS to increase the production of NO. 17 18 Thus, balance between eNOS and ET-1 in the pathophysiology of PE became an area of interest. However, there are still controversial reports and limited information regarding role of placental tissue ET-1 and eNOS expression in PE. The present study evaluated the expression of ET-1 and eNOS in normal and preeclamptic placentae. Moreover, there was still doubt whether the increased serum level of ET-1 found in PE was related to placental ET-1. The present study evaluated the serum levels of ET-1 as well as the expression of ET-1 and eNOS in normal and preeclamptic placentae.

## Methods

A cross-sectional study was performed in singleton pregnancies of women with and without PE ( *n* = 10 in each group) who were admitted to the North Okkalapa General and Teaching Hospital from February 2019 to February 2020. Individuals with a new onset of hypertension (systolic blood pressure ≥ 140 mmHg or diastolic blood pressure ≥ 90 mmHg) on or after the 20th week of gestation were categorized as having PE by obstetricians. 19 Normal pregnancy is defined as uncomplicated normotensive singleton term pregnancy from 37 to 40 completed weeks. Pregnant women with diabetes mellitus, liver or renal disease, cardiovascular disease or primary hypertension, infectious diseases (such as malaria, AIDS, and hepatitis B and C), and cases of multiple pregnancy were excluded.

Participation in the study was voluntary. The selection of subjects was based on their availability and willingness to participate. A thorough explanation about the study was provided to all patients, and written informed consent was taken. History taking and the physical examination were performed according to a pro forma. Blood pressure and proteinuria were measured in each visit throughout the pregnancy. An estimation of proteinuria was performed using the dipstick method in spot urine (urine dipstick reading ≥ 1+ [a concentration of 30 mg/dl] 19 (Urocolor™10 SD Inc, Korea). Personal data and detailed clinical history were also reviewed from previous records. The study was approved by the Ethics Review Committee of University of Medicine 2, Yangon.

Sample collection was performed on the day of delivery. About 5 ml of venous blood from the antecubital vein were collected under aseptic conditions 1 to 2 hours before delivery. Then, the blood was placed into a serum separator tube and allowed to coagulate at room temperature and centrifuged at ∼ 3,000 rpm for 20 minutes. Then, the serum was promptly separated into aliquots and stored at -80°C until the assessment of the levels of ET-1 by enzyme-linked immunosorbent assay (ELISA).

Placental tissues were collected from normal and preeclamptic women who underwent emergency or elective cesarean section immediately after delivery. Placental tissue samples (measuring ∼ 1.5 × 1.5 × 0.5 cm) were cut from the maternal side of placenta ∼ 1.5 cm away from margin and 1.5 cm away from insertion of the umbilical cord in sterile conditions. The samples were immediately placed into a 15-ml centrifuge tube and then transported on ice to the Common Research Laboratory at University of Medicine 2, Yangon. The placental tissues were rinsed with phosphate buffer saline (PBS) three times to remove as many blood clots as possible and stored at -80°C until the analysis the expression of ET-1 and eNOS by western blot.

Sodium dodecyl sulfate-polyacrylamide gel electrophoresis (SDS-PAGE) was used to separate target proteins. Approximately 0.01 g of placental tissue for each sample was homogenized with 250 µL of homogenizing buffer (radioimmunoprecipitation assay [RIPA] buffer: 0.5 M of ethylenediaminetetraacetic acid [EDTA] [solution]: halt protease and phosphatase inhibitor cocktail [100:1:1]) twice for 20 seconds. Then, the samples were centrifuged at 5,000 rpm for 20 minutes in a centrifuge refrigerated at 4°C to separate the supernatant and palette. A lithium dodecyl sulfate (LDS) sample buffer (Invitrogen, Waltham, MA, United States) with 8 M of dithiothreitol (DTT) (20:1) was prepared and mixed with tissue lysate (1:3 ratio). The samples were boiled in digital dry bath at 100°C for 5 minutes and stored at -80°C. The total protein concentration of 20 µg/well for each sample was loaded in 12.5% SDS-PAGE, and a 30-mA current was applied for protein separation. For membrane transfer, a semidry transfer system (ATTA, Japan) and an invitrolon 0.45 µm polyvinylidene fluoride (PVDF) membrane (Invitrogen) were used for membrane blotting at 200 mA for 60 minutes. Then, the membrane was blocked to prevent non-specific binding with 3% skimmed milk. The primary antibody solution was prepared by mixing 3% skimmed milk dissolved in 1X tris-buffer saline 1X-TBS) and anti-ET-1/anti-eNOS antibody (Invitrogen) in a dilution of 1:1,000 and stored at 4°C overnight for antigen-antibody reaction. After overnight incubation, the membrane was rinsed with 1X-TBS with Tween 20 (1X-TBST) 4 times on a mechanical shaker (twice for 5 minutes, and twice again for 10 minutes). The secondary antibody solution (3% skimmed milk dissolved in 1X-TBST and anti-rabbit immunoglobulin G (IgG)/anti-mouse IgG [Invitrogen]) was prepared in a dilution of 1:10,000 and incubated with the membrane for 1 hour at room temperature. After that, the membrane was once more rinsed with 1X-TBST 4 times. Then, it was rinsed twice with the 1X-TBS solution. The detection of protein band was performed with the Pierce ECL Plus Western Blotting substrate solutions A and B (in a 40:1 ratio) (Thermo Fisher Scientific, Waltham, MA, United States) and incubated for 1 minute. The protein band was detected by the X-ray cassette in the dark. The relative expression of placental eNOS was determined by the western blot method, and the intensity of the protein band was calculated using the ImageJ software (National Institutes of Health, USA). The values were expressed relative to age-matched controls (fold change value > 1.0 indicates an increase in abundant relative to the control).

For the assessment of the levels of ET-1 in the placental tissue, placental tissues that had already been rinsed were mixed with PBS (1 g of placental tissue + 9 mL of PBS) and homogenized (twice for 20 s) in an ice bath. Then, the placental tissue lysates were centrifuged at 5,000 rpm for 20 minutes in a refrigerated centrifuge, and the resulting supernatants were collected in separated aliquots and stored at -80°C until being analyzed by ELISA. The levels of ET-1 in the serum and placental tissue were determined using an ET-1 ELISA kit (MBS761947, MyBiosource, San Diego, CA, United States).

Data was analyzed using the Statistical Package for the Spcial Sciences (SPSS for Windows, SPSS Inc., Chicago, IL, United States) software, version 16.0. The variables pertaining to the outcomes were expressed as medians and interquartile ranges (IQRs) and computed by non-parametric tests. The comparison of the ET-1levels in the serum and in the placenta, and the placental eNOS expression between normal and preeclamptic pregnant women was performed using the Mann-Whitney U test. The correlation studies were performed using the Spearman correlation coefficient. Values of *p* < 0.05 were considered statistically significant.

## Results

The general characteristics of participants of the present study are presented in Table 1 .

**Table 1:** General characteristics of the study subjects

	Normal pregnancy ( *n* = 10) (mean ± standard deviation)	Preeclampsia ( *n* = 10) (mean ± standard deviation)	*p* -value
Maternal age (years)	28 ± 4.3	28.5 ± 5.7	Not significant
Systolic blood pressure (mmHg)	114 ± 5.2	156 ± 13.5	< 0.01
Diastolic blood pressure (mmHg)	73 ± 4.8	100 ± 14.1	< 0.01
Gestational age at birth (weeks)	39 ± 1	36 ± 2	< 0.05
Fetal birth weight (grams)	3,070 ± 312.8	2,720 ± 644.4	Not significant

**Fig. 1:**
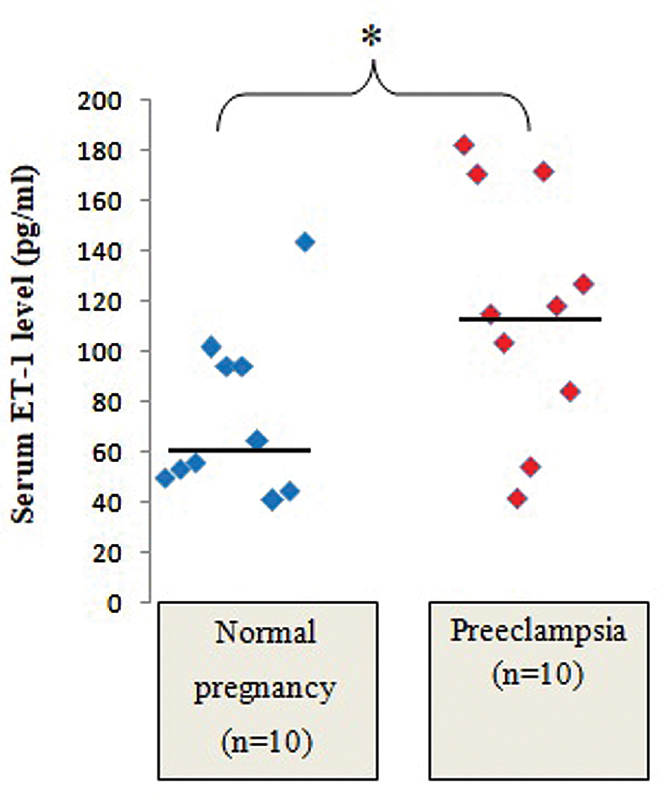
Comparison of serum ET-1 levels between normal and preeclamptic pregnant women. Abbreviation: NS, not significant. Notes: statistical test: Mann-Whitney U Test; the solid line indicates the median value.

**Fig. 2:**
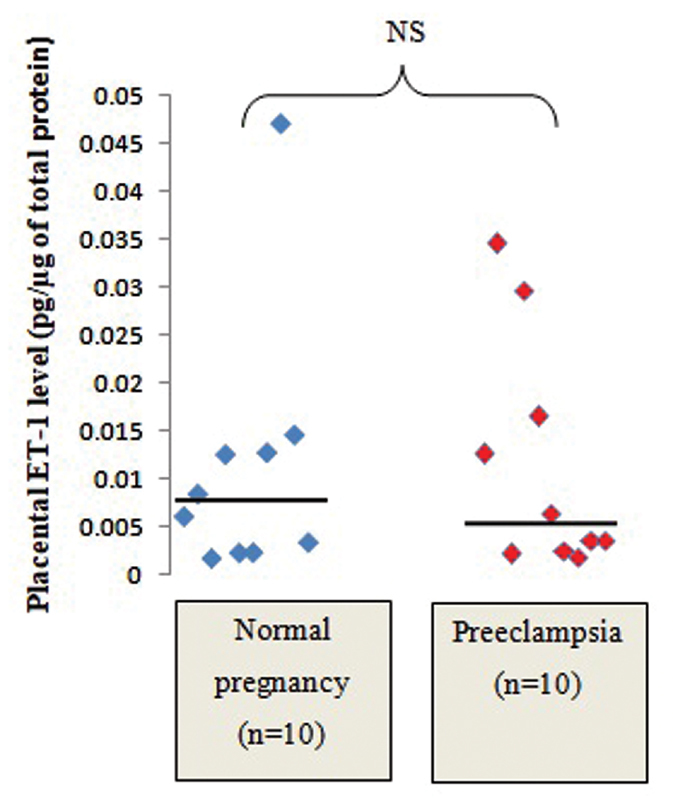
Comparison of placental ET-1 levels between normal and preeclamptic placentae. Abbreviations: N, normal pregnancy; PE, preeclampsia.

**Fig. 3:**
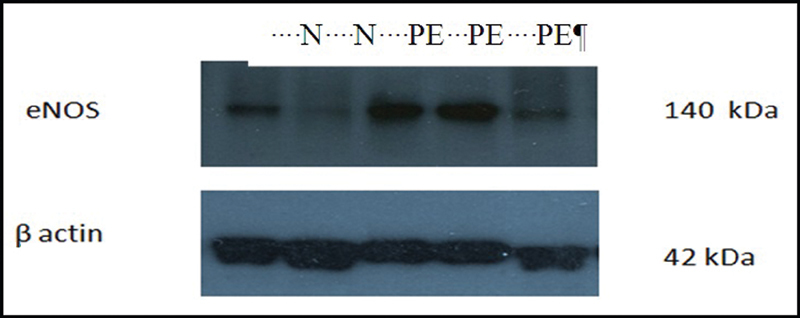
Placental eNOS expression in normal and preeclamptic placentae. Notes:* statistical significance ( *p* < 0.05); statistical test: Mann-Whitney U Test.

**Fig. 4:**
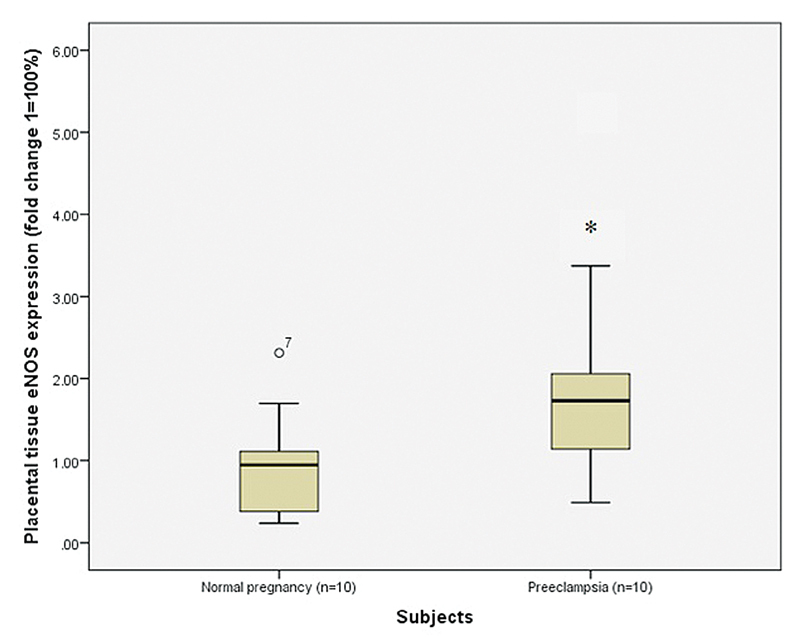
Comparison of placental eNOS expression between normal and preeclamptic placentae. Notes: * statistical significance ( *p* < 0.05); statistical test: Mann-Whitney U Test; the solid line indicates the median value.

The PE group had significantly higher sereum levels of ET-1 than the normal group (median: 116.56 pg/mL; IQR: 89.14–159.62 pg/mL; and median: 60.02 pg/mL; IQR: 50.89–94.37 pg/mL; *n* = 10; *p* < 0.05) ( Fig. 1 ), but there was no significant difference regarding the ET-1 levels in the placental tissue between normal (median: 0.007 pg/µg of total protein ; IQR: 0.002–0.0123 pg/µg of total protein) and preeclamptic placentae (median: 0.005 pg/µg of total protein; IQR: 0.003–0.016 pg/µg of total protein) ( Fig. 2 ). The expression of eNOS in the placental tissue was significantly higher in preeclamptic placentae than in normal placentae (median: 1.728; IQR: 1.229–2.042; and median: 0.945; IQR: 0.48–1.11 respectively; *p* < 0.05) ( Figs. 3 and 4 ). There was no significant correlation between the placental and serum ET-1 levels of both study groups ( Table 2 ). Neither there was a significant correlation between the ET-1 levels in the placental tissue and eNOS expression in both study groups ( Table 2 ).

**Table 2:** Correlation between the placental and serum levels of endothelin-1 (ET-1) of the study sample ( *n* = 20)

Correlation between	ρ (rho) value	*p* -value
Placental ET-1 and serum ET-1 levels in normal and preeclamptic pregnant women	−0.3	0.19
Expression of placental ET-1 and placental eNOS in normal and preeclamptic pregnant women	0.28	0.23

Note: Statistical test: Spearman correlation coefficient.

## Discussion

In the present study, we selected preeclamptic women and age-matched controls since aging is likely to have an effect on NO synthesis and activity. 20 Hence, there was no significant difference in maternal age in the whole sample. The mean gestational age at birth of the PE group (36 ± 2 weeks) was significantly lower than that of the normal group (39 ± 1 weeks) ( *p* < 0.05), since birth is the definite treatment to relieve the symptoms of PE. Even though eNOS expression and the level of NO increase with gestation until third trimester and return to prepregnancy levels postpartum, 21 the plasma levels of nitrite and nitrate are independent of the duration of gestation during the third trimester of pregnancy. 6 In the present study, all the pregnant women were in their third trimester, so it was unlikely to affect the NO level. The mean fetal birth weight of the PE group (2720 ± 644.4 g) was lower than that of the normal group (3070 ± 312.8 g) but without statistical significance.

In a normal healthy pregnancy, cytotrophoblasts originated from the fetus migrate and invade the maternal spiral arteries, undergoing a transformation into an endothelial-like cell. This remodeling converts the normally low-capacitance, high-resistance maternal spiral vessels into high-capacitance, low-resistance vessels. In the preeclamptic placenta, due to the failure to remodel, insufficient blood flow to the uteroplacental unit results in poor placentation. 22 This poor placentation causes repeated periods of placental hypoxia and reperfusion injury, resulting in endothelial damage. On the other hand, hypoxia increases the production of ROS, leading to oxidative stress. 23 Consequently, vascular endothelial damage and oxidative stress in the placenta increase the production of placental factors such as soluble fms-like tyrosine kinase-1 (sFlt-1) and soluble endoglin (sEng), inflammatory cytokines (tumor necrosis factor alpha, TNF-α), and agonistic autoantibodies to the angiotensin II type-1 receptor (AT1-AA). 24 25 These placental factors enter the maternal circulation and cause generalized maternal vascular endothelial dysfunction, leading to increased production of vasoconstrictors (ET-1). 9 Moreover, it was also noted that ET-1 was positively correlated with anti-angiogenic factors (sFlt-1and sEng) released by the placenta. 9 26

Another important mechanism is the inflammatory and autoimmune responses to placenta ischemia. The production of the agonistic autoantibodies to the AT1-AA seems to be causally related to placental ischemia through the induction of TNF-α. 27 28 Consequently, angiotensin II stimulates ET-1 expression. 29 Consistent with the previous studies, 8 9 26 the present study also showed that the serum levels of ET-1 were significantly higher in the PE group than in the normal group. Since serum ET-1 and the severity of PE symptoms are related, 11 the assessment of the serum levels of ET-1 could be considered an additional investigation in PE patients.

The present study determined placental levels of ET-1 in the maternal surface of placental tissue homogenates from normal and preeclamptic pregnant women by ELISA. The ET-1 levels in the placental tissue were very low in both study groups. There was no significant difference in tissue ET-1 concentrations between normal (median: 0.007 pg/µg of total protein; IQR: 0.002–0.0123 pg/µg of total protein) and preeclamptic placentae (median: 0.005 pg/µg of total protein; IQR: 0.003–0.016 pg/µg of total protein). Consistent with the findings of present study, there is much evidence of no significant differences in preproendothelin-1 mRNA gene expression by northern-blot analysis, 15 16 generation of ET-1 precursor by specific radioimmunoassay analysis, 14 and mature ET-1 by ELISA 8 between normal and preeclamptic placentae.

Contrary to the result of present study, some studies have reported that placental ET-1 expression was significantly higher in preeclamptic placentae than in normal placentae, 12 13 whereas other studies have reported that ET-1 mRNA expression was significantly lower in preeclamptic placentae than in normal placentae. 14 In these studies, placental ET-1 mRNA expression was determined by northern-blot analysis, 12 real-time polymerase chain reaction (RT-PCR) 14 and western blot. 13 In a study by Irtegun et al. 13 using the western blot method, the placental ET-1 expression in normal pregnancies was not at a detectable level, but preeclamptic placental ET-1 expression was high enough to detect. Likewise, in the present study, tissue ET-1 expression was too low to be detected by the western blot method in both normal and preeclamptic placentae. However, it could be detected by ELISA in the present study. Taking into consideration both the present and previous studies, discrepancies between methods of analysis might yield controversial findings. It is important to consider the methodology used to detect and interpret ET-1 levels to establish the diagnosis and severity of the disease.

In the present study, placental ET-1 level was not significantly different between normal and preeclamptic placentae, while serum ET-1 level was significantly higher in preeclamptic pregnant women compared with normal pregnant women. In agreement with this finding, Bernardi et al. 8 demonstrated that there was a significant difference in serum ET-1 levels but not placental ET-1 levels between normal and preeclamptic pregnant women. In the present study, the placental ET-1 level was not correlated with the serum ET-1level in any of the subjects, a finding also observed by Bernardi et al. 8 According to the findings of the present study together with those of the study by Bernardi et al., 8 it can be assumed that serum ET-1 rather than placental ET-1 plays a role in the pathogenesis of PE. A significantly high level of serum ET-1 in PE might be produced from the maternal vascular endothelium rather than the placenta. It can be suggested that targeting serum ET-1 could be used to determine an accurate value for the diagnosis and prognosis of PE, but this has to be validated before any implementation in the clinical practice.

In the present study, the relative eNOS expression was significantly higher in preeclamptic placentae than in normal placentae ( *p* < 0.05). In line with this finding, Myatt et al. 30 showed increased immunostaining for eNOS in stem villous cells of the fetoplacental unit in PE pregnancies compared with control pregnancies. Likewise, Napolitano et al. 12 found a significantly high level of eNOS mRNA expression in trophoblastic cells of preeclamptic placentae compared with the trophoblastic cells of normal placentae. In an in vitro study, Baker et al., 3 investigated NO production and eNOS activity in the endothelial cell line from a bovine coronary microvessel, and they reported that NO production and eNOS activity were significantly higher in endothelial cells exposed to plasma from the preeclamptic women than in endothelial cells exposed to plasma from normal pregnant women. 3 They concluded that there might be a factor or factors in preeclamptic plasma which induced NO production and eNOS activity in endothelial cells.

A vasoconstrictor, when its levels are increased, ET-1 regulates itself through a negative feedback mechanism, acting on ET-1 receptor B (ET _B_ 1) in maternal vascular endothelial cells to activate eNOS to increase the production of NO, a vasodilator. 17 18 Taking into account all of these findings and those of Baker et al., 3 one can be assume that increased eNOS expression in the placental tissue of preeclamptic placenta in the present study might be due to exposure of maternal serum containing ET-1.

Contrary to the result of present study, other studies 31 32 found no significant difference in eNOS immunostaining in syncytiotrophoblasts from preeclamptic placentae compared with normal placentae. Another in vitro study, conducted by Wang et al., 5 determined eNOS expression in endothelial cells isolated from normal and preeclamptic pregnancies, and reported that a significant reduction in eNOS expression was also noted in preeclamptic placentae. The underlying reason for the reduced eNOS expression might be the increased oxidative stress in PE. Increased levels of free radicals (reactive oxygen species, ROS) cause oxidation of tetrahydrobiopterin (BH _4_ , which is required for eNOS coupling) to trihydrobiopterin (BH _3_ ), consequently resulting in eNOS uncoupling.

Placental ET-1 mRNA expression is upregulated in early-onset PE (gestational week [GW] ≤34) and downregulated in late-onset PE (GW > 34) compared with age-matched normal pregnancies. However, the mRNA changes were not found at the protein level in all subjects in a study by Dieber-Rotheneder et al. 14 In the present study, all preeclamptic participants had late-onset PE, and the ET-1 protein level was not significantly different between normal and preeclamptic pregnancies. There might be a complex regulation of the endothelin system, and further molecular studies will be needed.

Under normal physiological conditions, serum NO and ET-1 are natural counterparts in vasculature function and may remain in a delicate balance. 1 It has been reported that NO antagonizes the ET-1 pathway via several mechanisms, including expression, 33 release, 34 receptor interactions, and second-messenger signaling systems 35 in the vasculature. On the other hand, ET-1 acts on the ET _B_ 1 located on the vascular endothelium to stimulate eNOS, resulting in increased NO production. 17 18 Accordingly, in the pathophysiological condition of diminished NO bioavailability, like in PE, a compensatory increase in NO can be induced by ET-1 via stimulation of eNOS. In support of this concept, a previous study conducted by Napolitano et al. 12 reported that exogenous ET-1 upregulated eNOS mRNA expression in cultured trophoblastic cells in both preeclamptic and normal trophoblast cell cultures. However, the present study showed no significant correlation between placental eNOS expression and placental ET-1 level or serum ET-1 in both normal and preeclamptic women. In the present study, we found no relationship between ET-1 levels in the placental tissue and eNOS expression, and this might be due to ROS as a product of oxidative stress, which might play an important role in eNOS uncoupling. Another reason might be the relative small sample size for the correlation analysis.

## Conclusion

The serum levels of ET-1 in preeclamptic pregnant women were significantly higher than those of normal pregnant women. Since exposure of maternal serum containing ET-1 to placental vascular endothelial cells stimulates eNOS expression through ET _B_ 1 on placental vascular endothelial cell, the relative eNOS expression was significantly higher in preeclamptic placentae than in normal placentae. The levels of ET-1 in placental tissue were very low and not significantly different between the study groups. Moreover, the placental ET-1 level was not significantly correlated with the serum ET-1 level in normal and preeclamptic pregnant women. We conclude that serum ET-1 rather than placental ET-1 may play a more important role in the pathogenesis of PE.
